# A protocol for a cluster randomized trial comparing strategies for translating self-management support into primary care practices

**DOI:** 10.1186/s12875-018-0810-x

**Published:** 2018-07-24

**Authors:** W. Perry Dickinson, L. Miriam Dickinson, Bonnie T. Jortberg, Danielle M. Hessler, Douglas H. Fernald, Lawrence Fisher

**Affiliations:** 1 Department of Family Medicine, University of Colorado School of Medicine, 12631 E. 17th Ave., Mail Stop F496, Aurora, CO 80045-0508 USA; 2 Department of Family and Community Medicine, University of California, San Francisco, San Francisco, CA USA

**Keywords:** Primary care, Type 2 diabetes mellitus, Practice facilitation, Self-management support, Patient-centered medical home, Psychosocial factors, Interactive technology

## Abstract

**Background:**

Advanced primary care models emphasize patient-centered care, including self-management support (SMS), but the effective use of SMS for patients with type 2 diabetes (T2DM) remains a challenge. Interactive behavior-change technology (IBCT) can facilitate the adoption of SMS interventions. To meet the need for effective SMS intervention, we have developed Connection to Health (CTH), a comprehensive, evidence-based SMS program that enhances interactions between primary care clinicians and patients to resolve self-management problems and improve outcomes. Uptake and maintenance of programs such as CTH in primary care have been limited by the inability of practices to adapt and implement program components into their culture, patient flow, and work processes. Practice facilitation has been shown to be effective in helping practices make the changes required for optimal program implementation. The proposed research is designed to promote the translation of SMS into primary care practices for patients with T2DM by combining two promising lines of research, specifically, (a) testing the effectiveness of CTH in diverse primary-care practices, and (b) evaluating the impact of practice facilitation to enhance implementation of the intervention.

**Methods:**

A three-arm, cluster-randomized trial will evaluate three discrete strategies for implementing SMS for patients with T2DM in diverse primary care practices. Practices will be randomly assigned to receive and implement the CTH program, the CTH program plus practice facilitation, or a SMS academic detailing educational intervention. Through this design, we will compare the effectiveness, adoption and implementation of these three SMS practice implementation strategies. Primary effectiveness outcomes including lab values and evidence of SMS will be abstracted from medical records covering baseline through 18 months post-baseline. Data from CTH assessments and action plans completed by patients enrolled in CTH will be used to evaluate practice implementation of CTH and the impact of CTH participation. Qualitative data including field notes from encounters with the practices and interviews of practice personnel will be analyzed to assess practice implementation of SMS.

**Discussion:**

This study will provide important information on the implementation of SMS in primary care, the effectiveness of an IBCT tool such as CTH, and the use of practice facilitation to assist implementation.

**Trial registration:**

Registered with ClinicalTrials.gov – ClinicalTrials.gov ID: NCT01945918, date 08/27/2013. Modifications have been updated.

## Background

Most patients with type 2 diabetes mellitus (T2DM) in the U.S. receive diabetes care in primary care settings, which are undergoing rapid transformations due to the need to improve quality and decrease costs. The Patient Centered Medical Home (PCMH) and the Chronic Care Model (CCM) are complementary clinical intervention frameworks that are commonly employed to support better T2DM outcomes in primary care [1–7]. Self-management Support (SMS) is a core component of both the PCMH and CCM, and focuses on the central role of patients in managing their illness by engaging with and adopting healthy behaviors that promote optimal clinical outcomes [6, 8–10]. SMS typically targets improvements in medication adherence, diet, exercise, and other risk-related behaviors; all of which are crucial for maintaining good glycemic control and reducing the risks of diabetes-related complications. Despite its recognized importance, SMS programs for diabetes continue to demonstrate limited effectiveness and sustainability in the real world of primary care [11, 12]. Primary-care physicians have been unable to comprehensively and consistently address diabetes self-management within an efficient and systematic SMS framework for several interrelated reasons: they are often overwhelmed by competing demands, poorly trained in assessing and intervening with health behavior change, lack practice systems for implementing change and quality improvement, and receive inadequate reimbursement for time spent in SMS activities [13–16].

Few tools are available to assist practices with self-management support. Interactive behavior-change technology (IBCT) can facilitate the adoption of crucial SMS interventions in primary care for patients with diabetes and related health risk behaviors [17–20]. Compared with traditional, unstructured programs, technological options for delivery have the advantage of increased convenience and accessibility, and may provide individualized support and resources necessary for initiating and maintaining healthful lifestyles, especially when they include non-automated options to address patient preference and permit patient tailoring [19–22] There is strong evidence that Internet-based programs can effectively promote health behaviors to support diabetes self-management, [23] such as healthful eating/weight management, [24–27] increasing physical activity, [28–30] reducing depression symptoms, and smoking cessation [31, 32]. Multiple randomized trials have been conducted using IBCT programs for diabetes self-management with positive results [33, 34]. However, most current IBCT self-management programs contain several limitations to translation into primary care settings: [35, 36] they are largely informational, they require high literacy, they are limited to simple health-risk assessment without goal setting, action planning or follow-up, they fail to provide physician decision support, and they do not emphasize patient-physician collaboration. Furthermore, most are exclusively automated and do not take into account the preference of many patients and clinicians for different modes of assessment and intervention.

Connection to Health (CTH) is a comprehensive, evidence-based SMS program that supports behavior change through IBCT. The CTH logic model is informed by social-cognitive [37–39] and social-ecological [40–42] theories and is inclusive of the evidence based principles for implementing SMS in primary care [43]. Multiple intervention components work together to promote enhanced, tailored diabetes management, which is linked to positive health outcomes [44–46]. Patients complete a CTH assessment that covers multiple issues related to diabetes and co-morbid conditions using state-of-the-art measures, each with cut-points defining a flagged area for concern. Patients receive an immediate summary, along with profiles from prior assessments to denote change over time. Patients are asked to review the summary and identify areas to discuss with their care team in preparation for making an action plan. A parallel report is prepared for the clinician that also includes decision support tools and options for the clinician for each flagged area on the profile, all to assist in beginning a conversation with the patient about specific problems and how they might be addressed. Action planning plays a central role, and includes goal setting and problem solving [47] through an automated, web-based action planning program. CTH also includes patient resources and tips to improve diabetes management. CTH is avehicle for structuring and guiding a time-effective clinical conversation between patient and the care team, enabling clinicians and patients to regularly assess, monitor, and intervene with self-management issues using a patient-centered approach that allows for practice and patient tailoring and encourages patient/care team interaction.

The simple availability of effective IBCT tools like CTH does not assure their successful implementation [15, 16, 48–50]. Primary care practices are experiencing multiple pressures to see a large number of patients, to provide improved care, and to do so with very constrained reimbursement. Practices have few mechanisms to integrate new programs into routine care, which can exert major pressures on practice operations – even small changes can have substantial consequences that limit their effectiveness and sustainability [15, 16, 48–50]. Adoption and implementation of new care programs varies across practices based on practice characteristics, including practice culture and change capacity, practice size, location (rural vs. urban), previous change experience, and decision-making style [13, 14, 51].

Practice facilitation has been effective in assisting practices in implementing organizational changes such the CCM and PCMH [52–58]. A facilitator can assist a practice in tailoring a new program to fit their unique practice situation, resources, and culture, improving its implementation and its sustainability over time. Practice facilitators use a motivational interviewing approach in assessing and increasing key stakeholders’ motivation for change. The practice facilitation intervention impacts the practice’s capacity for change through forming a quality improvement team, [59] training the team in quality improvement techniques including the use of Plan-Do-Study-Act (PDSA) for rapid cycle change, planning and implementing work flow and other changes, and identifying and working to overcome barriers. The quality improvement team consists of a diverse group of representatives from the various clinician and staff roles in the practice and meets regularly to plan the adoption and implementation of programs like CTH.

This study has been designed to promote the translation of SMS into primary care practices for patients with T2DM by combining two promising lines of research; specifically, testing the effectiveness of CTH for patients with T2DM in diverse primary care practices and evaluating the impact of practice facilitation to enhance uptake and maintenance of the intervention. Our specific aims for this project are:

1. To conduct a cluster randomized trial to examine the reach, effectiveness, adoption, implementation, and maintenance (RE-AIM) of CTH for patients with T2DM in primary care practices.

2. To determine the incremental benefit of brief targeted practice facilitation on the implementation of CTH in diverse primary care practices.

3. To identify key practice characteristics (e.g., practice size, organization, setting, and level of experience with practice redesign efforts) that affect CTH RE-AIM outcomes.

## Methods

### Design

**Fig. 1:**
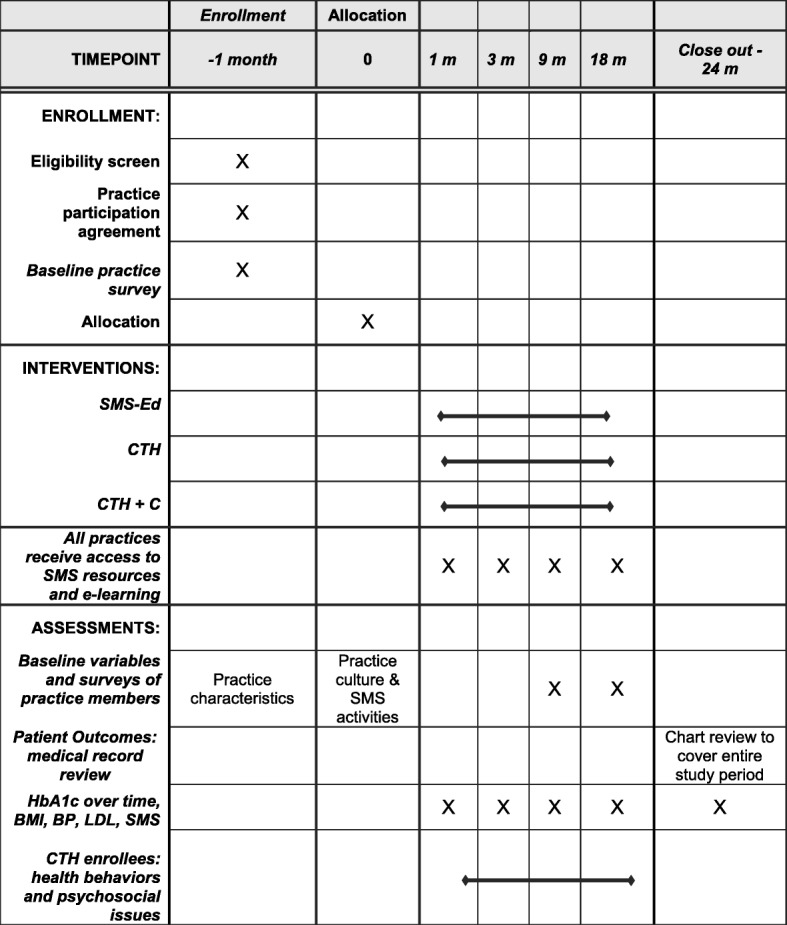
Timing of Enrollment, Interventions, and Assessments

To address these aims, we will undertake a three-arm, *cluster-randomized trial* to evaluate the reach, effectiveness, adoption, implementation, and maintenance of CTH for patients with T2DM in diverse primary care practices (Fig. 1). Primary care practices will be randomly assigned to receive and implement the CTH program (CTH), the CTH program plus practice facilitation (CTH + F), or an SMS academic detailing educational intervention (SMS-Ed). Through this design, we will compare the effectiveness, adoption and implementation of these three SMS practice implementation strategies. To assure maximum generalizability, we will recruit a diverse group of practices in Colorado and California. We have chosen this design in order to examine the incremental effect of both CTH (CTH vs. SMS-Ed) and practice facilitation (CTH vs. CTH + F) on SMS outcomes, compared to an academic detailing educational intervention. Blinding of practice participants or study team is not possible.

#### Sample

We will recruit 36 primary care practices, 18 each in Colorado and California in the United States, with equal numbers randomized to each of the three arms in each state. Inclusion criteria are family medicine or general internal medicine practices with a minimum of 80 patients with T2DM, with all clinicians agreeing to participate. We will recruit a diverse set of practices of various sizes and organizational structures (such as private, system-owned, and safety net practices, date of first enrolment 11/14/2013). Balanced study arms are essential to the success of cluster randomized trials. Imbalanced study arms can adversely affect study interpretability and potentially weaken causal inference [60–63].

Covariate constrained randomization procedures have been demonstrated [64, 65] to be effective for ensuring acceptable study arm balance in cluster randomized trials. Using this procedure, all possible randomizations will first be generated within each stratum (Colorado or California). Next, standardized baseline variables for all clusters will be used to compute a balance criterion, which is the sum of squared differences between treatment arms across all selected variables; the balance score is a measure of the *difference* between study arms, so low scores reflect less difference. For this three-arm trial, three balance scores will be computed for each randomization (arm 1 vs. 2, arm 2 vs. 3, and arm 1 vs. 3). The distributions of balance scores will be examined and a cutoff of the 25th percentile for all three will be used as a criterion for identifying the “optimal set.” From this set, a single randomization will be randomly selected using a random number generator and practices assigned to study arms by the project biostatistician.

### Interventions (see Table 1 and Fig. 1)

#### SMS education (SMS-Ed) arm

The SMS-ED arm serves as an attention control. Project staff will meet onsite with practice clinicians and staff members for two one-hour sessions to discuss what SMS is, why it is important, how primary care plays a role in this process, how others have approached it, and how it can be time and cost efficient for them to engage in SMS as part of standard diabetes care. Practices will have access to a website displaying general and local SMS resources, but will not have access to the formal CTH program. During this session, discussion of the implementation of these resources into the practice will be facilitated. No input will be provided regarding how unique practice characteristics might be utilized for more effective implementation of SMS, and CTH will not be introduced. Practices will be left to utilize these resources and to expand SMS activities as they see fit, without any further study input.

**Table 1:** Program Elements across Project Arms

Program Element	SMS-Ed	CTH	CTH + F
Connection to Health computerized intervention program	No	Yes	Yes
Technical assistance with CTH implementation	No	Yes	Yes
Basic instructions on use of CTH	No	Yes	Yes
Assessment of baseline SMS and diabetes care activities	Yes	Yes	Yes
Feedback of assessment and recommendations for practice	No	No	Yes
Generic SMS education	Yes	Yes	Yes
Generic website with SMS resources	Yes	Yes	Yes
Practice facilitation: • Improvement team meetings – 4 over approximately 3 mos. • Workflow revision to implement CTH • Email contacts, other assistance between improvement team meetings and after 3 months as needed • Ongoing feedback of data regarding CTH usage	No	No	Yes

#### Connection to Health (CTH) arm

Practices in this arm will receive instruction in the use of the full CTH program, including web-based resources, but will not receive any practice facilitation to assist with adoption and implementation. The number and length of visits to these practices will be the same as for the SMS Education Arm, plus an additional one-hour training session on the technical aspects of CTH. The content of the visits will center on the use of CTH as a way to implement SMS. Clinicians and selected staff members will be given hands-on experience using the system and will be provided with scenarios that will highlight the effective use of CTH as a tool for diabetes SMS. The practices will then implement CTH on their own, with additional technical assistance provided as needed.

#### Connection to Health plus facilitation (CTH + F) arm

This arm includes the same three one-hour training sessions as CTH, but adds short-term, focused practice facilitation. The active practice facilitation phase includes four meetings of the practice improvement team, scheduled for approximately 60 min each. The improvement team will consist of diverse representatives (generally 6–10) of the practice. The facilitator will assist the team in developing a CTH adoption plan and then help them identify small goals for rapid cycle change using the Plan-Do-Study-Act QI model. Active facilitation will last for approximately 3 months, followed by monthly calls by the facilitator to review data regarding the practice’s use of CTH. A brief “booster” facilitation can also be scheduled to address subsequent problems.

### Patient samples

#### Patient population perspective

Since allocation of patients occurs at the level of the practice in this trial, all *patients* within a practice will be assigned to the same treatment condition, regardless of the extent to which the individual patient uses the tools provided. Although the intervention can potentially impact the entire population of patients with T2DM, each practice in the CTH and CTH + F arms will selectively utilize CTH with patients, and some patients introduced to CTH in a practice will follow through with it while others will not. Therefore, practices in each of the two arms with CTH will have patients who were and were not exposed to CTH. To preserve intent-to-treat approaches and alleviate potential selection bias at the level of individual patient recruitment, [66–69] we will evaluate the effect of CTH by defining two overlapping patient samples in each practice. The first will be a random sample of all patients with type 2 diabetes mellitus, ages 21 and over diagnosed for a minimum of 12 months, whether or not they participated in CTH – the “Evaluation Sample.” This sample will enable us to examine the effectiveness of the intervention on the primary outcome variables in the intent-to-treat analyses. A second sample in each practice will be comprised of only those patients with diabetes who complete the CTH assessment – the “CTH Sample.” This sample will be used in “as treated” (per protocol) analyses. It should be noted that these two samples are not independent of each other; e.g., many in the CTH sample will be included randomly in the Evaluation Sample. Each sample, however, will be utilized to address different research questions.

#### Evaluation sample

Medical record reviews will be conducted on a random sample of patient who had received care in each practice for at least 1 year at baseline. Reviews will be conducted by research staff separate from the intervention team. The evaluator will be provided with a manual containing detailed instructions and will keep an audit log. A four-part data quality monitoring procedure will be employed consisting of use of a standardized abstraction protocol, extensive training in data abstraction, monitoring of Kappa values between the abstractors, and continuous quality improvement including periodic feedback to abstractors [70].

#### CTH sample

In addition to the evaluation sample described above, data from online CTH assessments, completed by those patients enrolled in CTH, will be used to evaluate the level of implementation of CTH in practices as well as the impact of CTH participation on patient SMS outcomes. The combined samples will result in medical record reviews on up to 1800 patients.

Overall, this study involves no greater than minimal risk to practice member participants or patients. The primary risk is loss of confidentiality. Chart audits are completed on site within the practice and no identifying patient information is removed from the practice. Practice member surveys are anonymous, and we only retain practice (not individual) identifying information. Data are reported only in aggregate. Procedures for minimizing risk are in place and any breeches reported to the governing IRB. Field notes from practice facilitators are monitored regularly by the study team for monitoring trial conduct and possible adverse events.

### RE-AIM evaluation

We will use the RE-AIM framework to guide our evaluation [71–77].

***Reach*** will be assessed by comparing the percentage of eligible T2DM patients for whom there is evidence on chart review that SMS was provided by the practice across the three arms. The reach of the use of CTH will be assessed in two ways in the CTH and CTH + F practices: (1) the percent of eligible T2DM patients with whom CTH is used by the practice (number of eligible T2DM patients enrolled in CTH / total number of eligible T2DM patients with diabetes in the practice), and (2) comparisons of patients who use CTH vs. patients who do not participate in CTH on the basis of key demographic and medical history information.

***Effectiveness*** will be measured in intent-to-treat analyses by several sets of outcome variables collected from the evaluation sample by medical record review. Additionally, evaluation of patient-level “as treated” effects of participation in CTH will be carried out by comparing outcomes for patients enrolled in CTH to those not enrolled on all measures common to CTH.

***Adoption*** will be assessed by the participation and representativeness of practices recruited for the study.

***Implementation*** will be assessed by (a) the extent to which various intervention components are delivered to participants compared to intervention protocol; (b) description of practice-specific modifications to the basic protocol that seem to be effective; and (c) CTH website usage data.

**Maintenance** of the CTH system will be assessed by the extent of continued patient use of the CTH system over the intervention period, as well as the percent of practices at the 18-month follow-up continue using the CTH program.

### Measures

#### Primary Effectiveness Outcomes

Will be obtained from the evaluation sample. HbA1c, LDL, systolic and diastolic blood pressure, and BMI will be abstracted from medical record reviews covering baseline through 18 months post-baseline. For each, the last measure prior to baseline will be used as the baseline measure.

#### Practice SMS: Process of care

The following elements will be assessed in medical record review: presence of a personal care plan with regular updating, evidence of collaborative goal setting, evidence of action planning around prioritized patient goals, evidence of collaborative problem-solving regarding the action planning process, use of community resources to assist in goal attainment, and evidence of ongoing monitoring of progress on identified goals. The chart audit form is available from the authors.

#### Practice Characteristics

Key practice characteristics will be used as covariates and potential moderators in analyses. Practice characteristics will be collected during the practice recruitment process, including level of quality improvement experience, level of PCMH implementation, practice size, setting (rural/urban), level/type of practice organization, baseline performance characteristics related to diabetes, percentage of minority patients in the practice, and percentage of Medicaid or uninsured patients. Additionally, practice members will be surveyed at baseline, 9, and 18 months to assess their perceptions of practice culture and provision of SMS to patients. Providers and staff who participate in surveys receive an information sheet explaining the evaluation purposes and procedures, and completion of the survey provided implied consent. Practice members included for interviews are provided with information by the study team and verbal consent obtained prior to proceeding with the interviews. Waiver of documentation of consent has been approved by the Institutional Review Boards.

#### Secondary effectiveness outcomes from the CTH assessment

Congruent with policy recommendations from the Society of Behavioral Medicine, [78] we use brief scales that are reliable, sensitive to change, and age appropriate in the CTH assessment [79]. These include: diet (intake of saturated fat, fruits and vegetables, salt, and sweetened beverages); [80–82] physical activity (frequency and duration of participation in vigorous, moderate, and walking activity, as well as “screen” time from the International Physical Activity Questionnaire); [83] medication adherence (number of days missed, reasons for missing); [84–86] alcohol intake (number of drinks in past week, number of times imbibed more than limit in past month); [87, 88] tobacco use (whether or not using tobacco, if so, how much;) [89, 90] depression (PHQ-8); [91, 92] and disease-related distress (modified from the Diabetes Distress Scale) [93, 94]. For patients who complete the CTH assessment more than once, we will be able to compare the results over time. For patients who only complete it once over the study period, we will use the data to help describe the sample of patients with whom the practices used CTH.

#### Data management

Standardized surveys and chart audit data collection tools and protocols with double data entry for paper forms are used throughout the project. Practice member surveys are administered at initial engagement with practice facilitation. Practice staff and clinicians could complete the survey either via paper copy or through an email link to REDCap. REDCap (Research Electronic Data Capture) is a secure, web-based application designed to support data capture for research studies. (REDCap is supported by supported by NIH/NCRR Colorado CTSI Grant Number UL1 RR025780.) Surveys are anonymous and no individual information was obtained. Data cleaning and coding are supervised by the project statistician. All core study team members will have access to the final dataset.

#### Power and sample size

For the evaluation sample, chart audits of 30 patients per practice will yield 360 patients per arm (1080 total). Assuming an intraclass correlation (ICC) of 1%, this sample size will provide > 80% power to detect a .25 SD difference between any two arms at follow-up or a medium linear trend effect [95, 96]. In a related study the SD for HbA1c was 1.7 and the ICC for patients within practice was < 1%. Thus, a .25 SD difference would translate to a final HbA1c difference of 0.43 between any two arms, adjusted for baseline differences. A similar effect size difference in systolic and diastolic blood pressure, LDL (all ICCs < 1) would translate to a final difference of 4.5 mmHg for systolic BP, 3.0 for diastolic BP, 8.8 for LDL.

#### Quantitative data analysis

For this cluster randomized trial, descriptive statistics will be computed for baseline patient and practice characteristics, initially testing for differences between: (1) different intervention arms and (2) CTH participants vs. non-participants. Patient-level covariates will be screened in bivariate analyses and included in multivariate analysis if they are related to the outcome at *p* < .2, differ between treatment arms, or are associated with dropout. In general, we will employ methods that utilize all available data, assuming ignorable missingness [97–100]. In the event normality assumptions are not met, we will use transformations to normalize distributions, ordinal or Poisson regression where appropriate, and/or the appropriate link function (e.g. logit link for dichotomized measures) [101–103]. We will employ intent to treat analyses using general (generalized) linear mixed models to incorporate data structures that are both hierarchical and longitudinal [66–69, 102, 104]. Hypothesis tests will be two-sided with alpha = .05 or *p* values reported. Goodness of fit statistics and model fitting diagnostics will be used to assess for influential points, outliers, overdispersion and heteroscedasticity and to evaluate alternative model specifications [101]. All statistical analyses will be performed using SAS version 9.2 (SAS Institute Inc., Cary, N.C.).

#### Qualitative data collection and analysis

Extensive qualitative data will be collected across the practices, including observational field notes and in-depth interviews done at baseline and at nine and 18 months from baseline. Field notes will be prepared by the facilitator and/or research staff after each substantive contact with the practice. These data will allow the qualitative analysis team to identify practice issues that impact the adoption, implementation, and maintenance of the SMS activities over time, including CTH in the CTH and CTH + F arms. The facilitator field notes from the improvement team meetings of practices in the CTH + F arm will provide especially rich data for assessing the responses to the facilitation intervention in those practices.

The qualitative analysis will be an iterative process, with the investigators going through cycles of reading, summarizing, and re-reading the data. The interpretative analysis will thus proceed iteratively through five phases of qualitative interpretive analysis described by Miller and Crabtree, [105] resulting in a rich interpretive summary of individual practices and themes running across practices.

#### Dissemination

Final practice assessments will be shared with each practice at the end of the study period. Core study team members will review all manuscripts and abstracts prior to submission. Results will be reported in peer reviewed publications.

#### Current status

Recruitment and data collection are complete as of October, 2017, and data analysis is in progress.

## Discussion

Approximately 70% of patients presenting to primary care practices in the United States have one or more chronic diseases. Primary care clinicians have a difficult time fitting multiple agenda items into brief patient visits and cannot easily meet the SMS needs of their patients with chronic conditions [106]. Approximately half of patients leave their primary care visit without an understanding of what their doctor told them, [106] and only 9% of patients report participating in any shared decision-making regarding their chronic condition [107]. In addition, average adherence rates for lifestyle changes are below 10% [108]. Thus, innovative, time-efficient, and engaging SMS methods need to be developed to assist patients and primary care clinicians and staff. Web-based programs and applications have shown promise for improvement in health behaviors, yet none facilitate patient engagement and shared decision-making between patients and their healthcare team. The use of CTH, with or without practice facilitation to support implementation, should improve SMS for patients with diabetes in primary care practices. This study will provide important information on the implementation of SMS in primary care practices, the effectiveness of an IBCT tool such as Connection to Health, and the use of practice facilitation to assist practices with implementing SMS.

## Abbreviations

CCM: Chronic Care Model
CTH + F: Connection to Health plus facilitation
CTH: Connection to Health
IBCT: interactive behavior-change technology
ICC: intraclass correlation
PCMH: patient-centered medical home
PDSA: plan-do-study-act
RE-AIM: reach, effectiveness, adoption, implementation, and maintenance
SMS: self-management support
SMS-ED: self-management support plus education (arm of the trial)
T2DM: type 2 diabetes mellitus
